# Violence prevention and incident documentation in elderly and special needs care institutions in southern Germany: results from semi-structured interviews

**DOI:** 10.3389/fpsyt.2026.1838036

**Published:** 2026-10-21

**Authors:** Miriam Kappe, Johannes Schobel

**Affiliations:** DigiHealth Institute, Neu-Ulm University of Applied Sciences, Neu-Ulm, Germany

**Keywords:** elderly care, qualitative research, special needs care, violence assessment, violence prevention, workplace violence

## Abstract

**Background:**

The likelihood of experiencing workplace violence is particularly high in the healthcare sector. Due to the lack of uniform guidelines for documentation, the number of unreported violent incidents is likely to be much higher. The elderly and special needs care sectors are particularly affected. Even though initial legal regulations for providing violence prevention concepts are in place, there are no uniform guidelines for documenting and handling incidents of violence.

**Aims:**

Our research focuses on presenting the current situation and providing a deep understanding of existing measures in violence prevention in elderly and special needs care institutions in southern Germany. Based on expert interviews the current situation on documenting and handling violent incidents is presented.

**Methods:**

This qualitative study is based on a purposeful sampling of 18 semi-structured interviews conducted with violence protection officers and institutional managers in elderly and special needs care. The analysis was carried out using qualitative content analysis according to Kuckartz.

**Results:**

The research provides practice-based insights of existing prevention measures in elderly and special needs care in southern Germany. Insights reveal that although some violence prevention concepts exist, they are not sufficiently implemented in practice. This leads to a fragmented image, especially when it comes to documentation. Interviews revealed that there are no uniform definitions of when an incident must be documented and reported. Also, managers cannot make concise statements about the frequency and context of incidents. Finally, considerable uncertainty was discovered about the severity categorization of incidents.

**Conclusion:**

This study uncovers the current situation of documenting violent incidents in elderly and special needs care institutions and reveals highly fragmented landscape when it comes to available digital applications, definition or classification of violence, or how to handle incidents.

## 1 Introduction

The German healthcare system faces major challenges, like a shortage of qualified employees, an increasingly aging society, and high employee turnover (1). A survey with 1,246 nursing employees at the Rhineland-Palatinate State Nursing Chamber in 2025 revealed that 35% of nursing employees often think about leaving their profession, and additional 35% occasionally do so (2). One possible approach to address these problems is to consider the job requirements. When asking about stressful events, in 2025, 37% of respondents cited aggressive clients or relatives, compared to 31% back in 2023 (2, 3). People working in healthcare are particularly at risk of experiencing workplace violence (4). The International Labour Organization defines workplace violence as follows: “The term “violence and harassment” in the world of work refers to a range of unacceptable behaviors and practices, or threats thereof, whether a single occurrence or repeated, that aim at, result in, or are likely to result in physical, psychological, sexual or economic harm, and includes gender-based violence and harassment” (5). Particularly when working with people who have dementia or with people with multiple disabilities, criminal responsibility is limited due to the lack of intent to act. Nevertheless, this study focuses on violence as factors contributing to workplace stress. It does not focus on determining criminal liability in the context of such violations. The question of guilt or criminal responsibility is not addressed and explicitly not focus of our investigation and the conducted study. “Violence” is referred to regardless of malicious intent. In healthcare settings, structural aspects, such as cramped working conditions, lack of places to retreat, high work pressure, and insufficient personnel, dramatically increase the risk of violent incidents (6). The situation is made even more difficult by the fact, that many institutions do not have proper violence prevention guidelines and lack sufficient resources for violence prevention (7). All together, these circumstances negatively affect employee satisfaction, particularly since safety is considered a hygiene factor and an important requirement (8). Even though the results cannot be directly applied to the setting of this study, there is evidence that workplace violence affects job satisfaction and the intention to leave. Among hospital doctors in China in emergency departments, a link between workplace violence and job satisfaction has been identified. Those who experienced more workplace violence had lower job satisfaction and were more likely to leave their jobs (9).

This study focuses on elderly and special needs care institutions. These are residential institutions and group homes for people with disabilities, as well as inpatient care. In Germany, regulations for implementing violence prevention concepts for healthcare providers in elderly and special needs care do not include any specific requirements regarding the content of these concept (10, 11). Accordingly, it can be assumed that there are major differences between the institutions in their measures. Existing guidelines often primarily focus on ensuring the quality of care, rather than prioritizing occupational safety for employees and clients. Often, introducing preventive measures are the first step of violence prevention. These measures are very important, as the likelihood of escalation increases significantly if training in violence prevention and de-escalation is inadequate (12). However, data is scarce concerning both the effectiveness of applied measures and the type and frequency of incidents (13). Even though there is no data available on these incidents, an increased risk of workplace violence must be assumed. Morken et al. investigated the occurrence of aggressive incidents in emergency primary care (14). The authors concluded that the main issues giving rise to these situations are unmet needs, involuntary assessment, and unsolicited touch. It is likely that these issues also play a critical role in elderly- and special needs care settings. Even though the context and the cause may be different, all three topics take on central importance in these settings. These institutions often care for people with multiple disabilities or dementia, having difficulties communicating their needs. It is mentioned that the frustration of not being understood or having one’s needs recognized can trigger aggressive behavior in such situations and environments.

Existing literature from German-speaking countries primarily deals with settings like hospitals or emergency rooms (15, 16), psychiatry (17) and the care for people with dementia (18). Often, existing documentation schemes are adapted to specific scenarios, record types and frequencies of violence. The literature on the current situation regarding prevention of violence, specifically in institutions for the elderly and special needs care is limited. At present, the issue can only be identified through individual studies with limited time frames and overly broad occupational groups or related fields. There are no government reporting or monitoring activities.

Vrablik et al. developed a framework based on the premise that workplace violence must be addressed at the micro level (employees and patients), the meso level (organization), and the macro level (justice and worldview) (19). Violence prevention is addressed from a strategic and organizational perspective. It therefore belongs to the meso level. It seeks to describe current measures for prevention and documentation within the cultural context of elderly and special needs care institutions. The goal here is to derive implications at the organizational level. Existing prevention and documentation measures should be reviewed and understood in order to develop concrete, practical starting points for standardized, centralized documentation.

This manuscript addresses following research questions:

RQ1: Current Situation

What measures for preventing and documenting incidents of violence are currently used in elderly and special needs care?

RQ2: Criteria for Classifying Incidents of Violence

What criteria are used to classify the severity of incidents of violence in the work of elderly and special needs care institutions?

To answer these research questions, data was collected on the current processes and regulations for dealing with incidents of violence. In addition, it was determined how and in what way incidents are currently documented, how protection against violence is organizationally represented, and which criteria are used to classify the severity of violent incidents.

This manuscript provides first insights into violence prevention in elderly and special needs care, revealing insights into measures that are commonly used in the practical work environment. Practice-based insights into existing concepts for prevention and documentation in elderly and special needs care are presented. The results provide an indication of when, how, and in what way individuals can document, report, and handle incidents of violence. This basis will enable incidents to be quantified more accurately according to their nature and context. In addition, relevant key indicators in the area of violence prevention in institutions are determined. The objective is to obtain information on how the documentation and monitoring of activities in violence prevention should be organized.

## 2 Materials and methods

As practice-oriented research, first the problems, needs and user contexts are identified (20), as violence is a complex and emotionally charged topic. In addition to the existing procedures, the goal is to gain contextual insights and understand prevailing opinions. For this purpose, a qualitative study-design with semi-structured interviews was conducted (21). Interviewees were experts on elderly and special needs care. The interview guide was developed in accordance with the recommendations of Kallio et al. To address the previously mentioned research questions, our interview guide covered four main topics: (1) Definition of Violence, (2) Documentation, (3) Incident Handling and (4) Incident Assessment. Additionally, demographic questions were asked. The guide was developed by the authors and tested iteratively by an internal research team. Close attention was paid to ensure that the wording was clear, concise and easy-to-understand. The interview guide was not provided prior to the interviewees. In addition, follow-up questions were asked when necessary to ensure better comprehensibility.

The main author has a background in healthcare management and is a certified quality management representative, focusing on topics in workplace research. The co-author is a research professor in the field of “Digital Medicine and Care” and director of the DigiHealth Institute at Neu-Ulm University of Applied Sciences. The interviews, as well as the subsequent transcription, anonymization, and coding, were conducted by the lead author as part of her doctoral dissertation and her work as a research assistant. The process was carried out under the close supervision of the second author. Any uncertainties or ambiguities were discussed within the three-person research team and resolved by majority vote. The research is being conducted as part of a project funded by the German Federal Ministry of Research, Technology, and Space (BMFTR) on the digital support of violence prevention strategies. One of the study’s objectives was to understand the needs and processes in practice and to be able to address them specifically within the framework of the project. The analysis clearly focused on providing digital support to the employees of the institutions. One of the interviewees was known to the researchers as a practice partner in the field of special needs care. This person was asked the same questions as all the other interview participants. Since agreements regarding the existing prevention strategy had already been reached within the context of the project, the responses contained less additional information than those from other participants. Nevertheless, the collected data was anonymized and analyzed in this form.

The data was evaluated using qualitative content analysis according to Kuckartz (22). The data analysis was conducted using the ATLAS.ti 26.0.2.33998 software. The first code category was determined by the research questions and interview guidelines. Additional categories were formed inductively based on the data material collected in the interviews. To analyze the current situation, 8 codes with a total of 41 sub-codes were created. Data was collected anonymously. The research procedure and all material was reviewed and approved by the Joint Ethics Committee of Bavarian Universities (GEHBa^1^) under vote GEHBa-202501-V-279.

After providing general information about the sample description, participants were asked about the definition and classification of “violence” in their institutions, and current measures for preventing and documenting incidents were reported. Participants were free to decide whether the interviews should be conducted in person or virtually. All participants chose to conduct the interviews virtually via Zoom so that they could participate flexibly from the office or from home. During the interview, only the interviewee and the interviewer were present. The interviews took place between June and August 2025 and lasted between 37 minutes and 1 hour and 8 minutes. The interviews were recorded using a voice recorder and initially saved as audio files. Transcripts were created on a local computer at Neu-Ulm University of Applied Sciences using the aTrain software (version 1.4.1.0). Because of the regional dialect, these transcripts were not entirely accurate, so they were manually revised and transcribed word for word after repeated listening to the audio files. In the process, all identifying characteristics—such as names, institution names, or demographic information that were not explicitly requested—were removed from the transcripts. For analysis, the interview transcripts were first read through in their entirety, and the relevant passages were highlighted. In the first step, the interview questions served as a deductive model for defining the main content categories. The material was coded, and the text passages assigned to the main categories were analyzed again to inductively generate subcodes. An overview of the codes and their usage, as well as the quotations used and their translations, can be found in the **supplementary material**. The audio files were deleted after transcription. The anonymized research data will be stored on a local server at Neu-Ulm University of Applied Sciences for 10 years as research data. After reaching a predetermined minimum total of 10 experts, an initial analysis was conducted. It turned out that the level of detail in the responses from individuals in elderly care differed from that of individuals in special needs care. Therefore, the data was analyzed separately to assess data saturation. Recommendations of Francis et al. were considered (23). Once the number of interviews per participant group reached five, we assessed whether the responses resulted in new codes. The analysis revealed that among participants in elderly care no new codes were generated after 5 interviews, while among participants in special needs care after 9 interviews. Two additional interviews were conducted with each group. Data saturation is assumed. All interviews were conducted in German. A translated excerpt from the interview guideline can be found in **Table 1**.

**Table 1:** Excerpt from the interview guide, translated from German.

Topic	Question
Sample Description	What is your job title?
Sample Description	How big is your institution?
Sample Description	What is your area of specialization?
Definition of Violence	What role does the issue of “violence” play in your institution?
Definition of Violence	How would you define violence?
Definition of Violence	How is violence defined within the institution?
Documentation	What triggers the documentation of an incident?
Documentation	What types of incidents are documented at your institution?
Documentation	What is the purpose of reporting incidents of violence at your institution?
Documentation	Does your institution have a policy for reporting incidents?
Documentation	Who reports and documents incidents of violence at your institution?
Incident Handling	What steps are taken after incidents of violence are reported?
Incident Handling	In what format is the data collected?
Incident Handling	Who uses the collected data afterward?
Incident Handling	Is there a clearly defined procedure that can be reviewed?
Incident Assessment	Does your institution use a standardized assessment framework when documenting incidents of violence?
Incident Assessment	How is the severity determined?
Incident Assessment	Who is involved in determining the severity level?
Incident Assessment	Who is responsible?
Incident Assessment	How are incidents of violence handled going forward?

### 2.1 Participants

The interviewees were recruited through the KompIGA^2^ project network and via an official list of special needs assistance institutions in the state of Baden-Württemberg. A purposeful sampling method was applied (24). Through specific inquiries and referrals individuals who have the most experience dealing with violence in institutions and are responsible for developing violence prevention concepts and the handling of incident at their institutions were identified. A total of 18 organizations that are not part of KompIGA were contacted. Of these, 14 were able to identify a potential contact person. The research project was presented after initially contacting them by phone and contact details for voluntary participation were provided. Upon interest, an explicit date for the interview was arranged. The participants received a detailed explanation of the study. They were informed that the study is part of a funded research project and is intended to develop a standardized approach to documenting and handling incidents of violence. All individuals who expressed interest in the study information participated in the interview.

Interviewees included violence protection officers, project employees working in the area of “violence prevention concepts” and de-escalation trainers. If a particular institution did not have an explicit violence protection officer, institution managers were also invited. In our case, this only applied to elderly care institutions. In addition, an interview with a violence protection representative from a large association was also included. The latter is responsible for creating guidelines on the topic of violence prevention for different institutions. Due to the nature of available contacts from our research network, this study is regionally limited to southern Germany. All interviews were conducted in German. All quotes provided in this manuscript have been translated into English for better understanding.

In total, 18 experts from 16 different institutions were interviewed. Seven of them work in elderly care, ten of them in special needs care. One person works in the headquarters and advisory office for a large association which includes members of both elderly and special needs care. This participant is in charge of approx. 80,000 clients. Since the person had previously worked in special needs care for a long time and most of their operational experience came from that setting, they were counted among the individuals from the special needs care institutions.

**Table 2** provides information on the sample. A few descriptive questions were asked, such as those concerning the size of the institution, its function and sector, as well as the organizational integration of violence prevention measures.

**Table 2:** Study participants.

ID	Profession	Primary focus	# employees	# clients
ID 1	Organizational Development	Elderly Care	1,500	1,200
ID 2	Project Development Violence Prevention Officer	Elderly Care	220	800
ID 3	Institution Manager	Elderly Care	45	35
ID 4	Head of Professional Development	Elderly Care	3,600	8,700
ID 5	Prevention and Violence Protection Officer	Elderly Care	2,900	6,000
ID 6	Home and Care Service Manager	Elderly Care	34	30
ID 7	Employee Central Quality Management	Elderly Care	600	600
ID 8	Quality and Complaints Management Officer	Special Needs Care	1,600	3,000
ID 9	Consulting and Service, Prevention Specialist	Special Needs Care	5,000	5,200
ID 10	Representative for Women’s- and Violence Protection (Association)	Elderly- and Special Needs Care	37,000	80,000
ID 11	Psychological Specialist and De-escalation Trainer	Special Needs Care	1,300	1,300
ID 12	Pastoral Care and Violence Prevention	Special Needs Care	600	600
ID 13	Employee Protection and De-escalation Trainer	Special Needs Care	180	200
ID 14	Specialist Service for Prevention and Crisis Intervention	Special Needs Care	2,000	5,000
ID 15	Violence Prevention Officer	Special Needs Care	3,000	3,000
ID 16	Prevention, Violence Prevention, and Sex Education	Special Needs Care	5,200	4,500
ID 17	Violence Protection Officer	Special Needs Care	200	80
ID 18	Admissions Management and Violence Protection Officers	Special Needs Care	1,600	3,000

## 3 Results

The results are divided into two main sections. First, the current situation, existing procedures for reporting, documenting and handling incidents are presented. Next, the criteria utilized for classifying the incidents are shown.

### 3.1 Current situation

To gain a better understanding of the domain, we asked general questions about existing prevention measures in their institutions.

The integration of violence prevention strategies in institutions varies largely. Sometimes, it is mainly handled by management. In bigger institutions, however, developing violence prevention concepts, its continuous review and adherence is part of a support unit, which most likely also is responsible for organizational development or equality. Violence protection officers are also responsible for the continuous development of these concepts. In some institutions, the topic of violence prevention is also handled by pastoral care providers or specialist services. Eleven of the participants also stated that they already have internal guidelines for dealing with incidents of violence.

The most frequently mentioned measures were training courses for different topics (i.e., sexual violence), and specific target groups (i.e., employees, clients). In addition, most institutions cover topics such as non-violent communication and de-escalation, self-defense, and dealing with certain medical conditions. Six institutions also stated that they had dedicated employees trained in de-escalation programs. This training also includes cultural and awareness-raising work within teams. Topics like “participation” and “self-determined participation” are also key training courses. Representatives from some special needs care institutions also stated that they offered such training, particularly with a focus on “social skills” and “sex education” for their clients. Half of the participants stated that they even have specific representatives responsible for such topics. Two institutions also mentioned external offices for consultation and reporting. Other factors relevant in the context of violence prevention mentioned include self-reflection, risk analysis, and the use of pagers to quickly alert nearby colleagues. Eleven of the interviewed institutions stated that they have a violence prevention concept in place.

In another part of the interview, we relate to the handling of incidents. Questions about existing documentation, the purpose of documentation, and the assessment frameworks used, were asked.

When discussing the current situation of documentation with experts, it became clear that in most institutions no standardized documentation of incidents exists. If a guideline for reporting and handling incidents is present, it usually involves a lot of manual paper-based work and is often not supported by specialized IT systems. Participants specifically mentioned manually transferring data in various document formats and copy/pasting from one document to another. Most interview participants stated that they rely on traditional paper-based forms for reporting incidents in their institutions. Sometimes, a digital version is also available as PDF or word document. In most cases, this reporting form is only used for serious incidents. At the same time, participants acknowledged that defining the severity of an incident is very difficult and highly subjective. One interview partner stated.

“[ … ] and then we also have a reporting obligation for serious incidents of violence. We searched for a very long time for a definition of “serious incidents” and were unable to establish any fixed criteria in our institution.” (ID 8, personal communication, author’s translation)

In general, the currently available documentation of violent incidents presents a very fragmented picture. For example, Person 3 stated:

“I make a brief note in my files if I have any suspicions. If it affects a client, it is always documented in the care report. [ … ] At the moment, we are still pursuing a dual approach. First, we have an electronic quality manual, every employee has access to. The incident form is posted there. You can also print this out. And then we have various team channels via Outlook. [The entire documentation] can also be accessed on the channel.” (ID 3, personal communication, author’s translation)

Even the definition of when an incident must be reported and documented is vague in most institutions. All client-related incidents, as well as challenging behaviors, are reported in the daily documentation at all institutions. Person 1 stated:

“In the case of residents, it is documented in the form of challenging behavior and, conversely, when violence is used against other clients. So [that includes] verbal abuse, kicking, biting, hitting—whatever clients do. But sexual advances are also part of it to a certain extent. For employees, the nursing service management does this in their own documents. In the [client-focused] documentary, the behavior of a caregiver should not be included.” (ID 1, personal communication, author’s translation)

Similarly, Person 4 reports:

“A distinction must be made between documentation in the client file and documentation in the complaints manual or critical incident book. We have a digital manual for quality management. There is a form to fill in digitally. However, it cannot yet be digitally signed and forwarded to respective person in charge. So, theoretically, it has to be printed out, signed, then it can be scanned again and mailed. Or it is thrown into the suggestion box, for example.” (ID 4, personal communication, author’s translation)

Person 12 stated that only incidents involving clients that go beyond pinching or spitting are documented.

“I’m not entirely sure about employees, but ultimately it ends up with the HR department and is documented on an external USB drive.” (ID 12, personal communication, author’s translation)

Half of the interview participants stated that they also rely on handwritten or digital documentation for specific cases. If necessary, these documents are also included in the employee file in HR department or added to the client’s record. Handwritten notes are most likely digitized sometime later (i.e., scanned) and attached to the record. Person 2 stated:

“At the moment, it is only accessible via a calendar linked to an email address that only I have access to, where I store notes. [ … ] It still needs a secure area that no one can access.” (ID 2, personal communication, author’s translation)

Finally, reports dealing with accidents are documented and sent to the (German) employer’s liability insurance association. This must be done if employees are clearly injured (i.e., cuts, bruises,…).

In general, our interviews revealed that documenting incidents serve various purposes in institutions: First, one key requirement is fulfilling regulatory and legal documentation obligations. For serious cases, it is mandatory to report the incident to the supervisory authority for residential care institutions and social welfare agency. Such obligations also exist within religious associations and supporting structures. In case an employee is injured, there are reporting and documentation obligations to the employers’ insurance association. At the same time, the documentation also serves as proof to external parties that potential risks have been identified and countermeasures were implemented.

Second, documentation serves the practical purpose of clarifying cases. This involves internal case communication within the emergency response team, employee discussions, case reviews, and deriving actionable strategies and countermeasures. Follow-up care is also an essential purpose of documentation. Even after the case has been closed, it should still be possible to contact employees and ask questions about any needs that may have arisen subsequently. Person 15 and 14 stated:

“We have found that it is helpful to write down the facts as quickly as possible. The longer it takes, the more blurred the whole incident becomes. So, this is then used for work and later for evaluation over the course of the year in order to determine frequencies and then draw conclusions for prevention. Once this is done, we will review the process again in two or three months’ time and assess the reporting chain in order to improve it on a regular basis.” (ID 15, personal communication, author’s translation)

“This means that I can follow up with my colleagues and ask them how they are doing and what they need at the moment – this serves to protect employees.” (ID 14, personal communication, author’s translation)

Third, is to protect those who were affected by the incident. Information about incidents is passed on to occupational safety in an anonymous way. Additionally, the documentation also serves as protection against possible long-term consequences. Person 13 stated:

“This means that if, for example, an employee has been hit in the abdomen and there are consequences weeks or months later, then this documentation naturally provides protection for the employee.” (ID 13, personal communication, author’s translation)

Finally, critically reflecting cases and deriving preventive measures were mentioned by interviewees. Documentation is used for reflection and optimization of general case handling itself. Anonymized data may also serve to identify potential risks, support redefining existing guidelines in place and may be used for educational purposes.

### 3.2 Criteria for classifying violent incidents

Clear, structured documentation requires objectification of the subject matter. Accordingly, we aim to identify characteristics of violent incidents by checking involved assessment framework or rating systems. The goal was to classify incidents according to their degree of intensity level or severity. This should serve to report incidents on the one hand, and on the other hand contribute to defining clear and comprehensible process steps depending on severity.

Interviews revealed that the institutions primarily classify incidents according to subjective perception. Person 10 stated:

“The assessment scheme is the perspective of the person concerned.” (ID 10, personal communication, author’s translation)

All participants stated that the individual assessment of the injured party is the main factor in assessing incidents. Interestingly, ten of the 18 participants stated that only subjective assessments are used in their institution. Person 11 stated:

“We have this list describing typical acts of violence. But now let’s return to our definition: If violence is defined in such a broad sense that the ‘denial of needs or desires’ can be experienced as violence by the persons concerned, then it is difficult to determine.” (ID 11, personal communication, author’s translation)

Participants explained that the institutions document and categorize incidents according to the existing form of violence. Person 17 describes:

“The reporting form lists the various forms of violence, but not their intensity.” (ID 17, personal communication, author’s translation)

Summarizing all interviews, they are often distinct between physical, psychological, sexual, spiritual, and/or cultural violence.

Some institutions rely on a traffic light system that categorizes incidents as “minor, moderate, or serious”. Others use a step system, which also consists of three escalation levels. Person 15 explained:

“We have three escalation levels [ … ]. One is for very minor offenses. Two and three are more serious injuries or have greater consequences. It is very difficult to draw the line between these two, and this is also a major learning field for employees.” (ID 15, personal communication, author’s translation)

Incidents classified as “green” (or “escalation level 1”) are usually those that are not documented. They can be resolved through direct intervention by employees and do not result in any visible injuries or require further communication or documentation. Person 5 stated,

“For green cases, the violence protection officer is not involved at the moment.” (ID 5, personal communication, author’s translation)

In the case of incidents classified as “yellow” (or “escalation level 2”), as well as “red” (or “escalation level 3”), the definition and distinction between both are significantly less clear. Participants stated that the degree of injury and the subjective assessment of the injured party must always be considered, as well as the external risk associated with the incident.

## 4 Discussion

This study responds to the call by Kuhlmann et al. to focus more closely on the protection of healthcare workers at the organizational level (25). Although their recommendations are directed at policymakers (macro level), they have implications at the organizational (meso) level. Given that there are no legal requirements in Germany to record and monitor violent incidents at the organizational level, yet statutory insurance providers and local authorities are primarily responsible for funding elderly and special needs care services, this is a gap. The findings identify areas for improvement in violence prevention at the organizational level and serve as a basis for developing practical measures to enhance documentation and monitoring.

The findings indicate that all participating institutions have implemented measures to enhance violence prevention. Organizational integration, however, varies significantly. While all surveyed institutions have established basic structures (i.e., dedicated staff positions), a more universal commitment to intensifying focus on violence prevention was observed across all interviewees. Notably, special needs care institutions already have detailed plans and comprehensive strategies in place to achieve objectives. In contrast, such measures remain less consistent in elderly care. This may be attributed to legislative development in Germany: Since 2021, special needs care institutions have been subject to a corresponding obligation (11), a requirement that is not yet reflected in elderly care. Vrablik et al. structure their model into prevention, response, and recovery activities (19). It became clear that even some of the existing violence prevention strategies operate at these three levels. When considering “prevention”, the examined institutions primarily focus on raising awareness and offering de-escalation training. At the “response” level, the policies outline initial procedures for handling incidents. In addition, some institutions already established contact persons and designated representatives to provide support in such situations. “Recovery” is addressed by using existing data to develop response strategies. In addition, incident data is shared with the occupational safety department and used to evaluate incident handling and the violence prevention concept.

Although violence prevention strategies provide a solid foundation, corresponding documentation of incidents remain fragmented. Although all participating institutions reported that incidents are recorded within their daily routine documentation, the current data management strategy remains decentralized. More specifically, incident records are typically stored in individual client files, rather than being aggregated in a centralized repository. Consequently, this hinders the systematic analysis of incident trends and prevalence. Here, too, it becomes clear that violence prevention is still largely viewed at the individual level in the institutions’ approaches. Incidents of violence are treated as isolated cases involving specific patients and employees. Each incident must be assessed and addressed individually. However, focusing solely on the micro level is not sufficient (19).

In the interviews, a significant discrepancy between the idealized protocol and practical documentation of incidents could be found. While most institutions theoretically mandate that “every incident” should be recorded, the lack of objective assessment criteria often leaves the definition of violence to subjective judgment of the individual. Regarding the definition of violence, most participants agreed that the classification of an event is up to the people involved (i.e., victim). This high degree of subjectivity and lack of objective assessment criteria prevents incidents from being handled in a structured manner. It follows from this that not only the authority but also the full responsibility for handling an incident of violence lies with the individuals involved. When it comes to the question of “when” an incident should be documented, the answer “ideally, every incident should be documented” remains correspondingly vague. These results are consistent with the statements by Schlup et al. (12), suggesting that a lack of a reporting culture (26) and the fear of negative consequences (27) contribute to inadequate documentation or deficits. In addition, the interviews made it clear that reporting and dealing with incidents of violence can also trigger subconscious inhibitions (i.e., “role conflict”) among employees. When employees experience violence, they usually transition from a caregiver to a victim in need of help and assistance. This transition could trigger subconscious inhibitions and feelings of guilt, as the need for help contradicts their professional self-image.

When discussing the assessment frameworks already in use, a significant inability among institutions to define comprehensible criteria for the objective classification of an incident was revealed. Arguably, this may be also related to the absence of an objective and standardized definition of “violence” within the sector. In some institutions, incidents are scaled into a three-tier system level. “Requires documentation and handling” is always the highest level, although the exact definition of these levels could not be clarified by interviewees. Some argued that this level involves incidents requiring the involvement of legal authorities, or executive board members. Yet, they could not provide specific triggers for such escalation. Participants stated that the current classification of incidents relies on *ad-hoc* consultations among involved experts. Consequently, the decision of whether and how to report, document, and handle an incident rests entirely on individuals. This lack of standardization is problematic, as it imposes a significant burden of responsibility on employees. Simultaneously it increases the risk of inconsistencies or inappropriate case processing, rendering a structured and reliable reporting system impossible to achieve.

Comprehensive framework conditions that contribute to classification or non-classification were also specified. Several participants stated that this also depended on the organizational culture. In some environments, an “aggressive tone” becomes normalized as part of daily work, resulting in a “sensitization” or “habituation” to violent situations where employees become desensitized to lower-level violence. In this context, Person 8 stated:

“[ … we noticed] that it really depends on what the employees in this particular residential institution are used to. If they experience such behavior on a daily, or hourly basis, then an incident is not considered reportable or even serious by these employees. [In another institution], this incident would be considered quite serious by colleagues.” (ID 8, personal communication, author’s translation)

This statement itself is quite alarming, as it implies that people who are accustomed to a violent environment are less likely to document and deal with violence. However, becoming accustomed to the circumstances should not influence the assessment, recording, and handling of violent incidents.

This issue is also addressed by Vrablik et al. (19). They argue that the normalization of workplace violence in healthcare depends on cultural perception, thereby classifying it as a macro-level issue. Upon closer examination, however, a paradox emerges. On the one hand, workplace violence in healthcare should be made more visible and recognized as a societal issue. On the other hand, institutions struggle to identify incidents of violence. A purely criminal law perspective (macro level) appears insufficient due to the perpetrators’ frequent lack of criminal responsibility. A determination that lies not within the discretion of institutions. The current approach of defining violence at a purely individual (micro) level is also insufficient, as it leads to a higher tolerance for violence due to the apparent normalization of such behavior. This reduces the likelihood that incidents of violence are reported and documented. Consequently, these incidents also become less visible. They are normalized not only by the victims themselves (micro level), but also by the organizations (meso level), and, ultimately at the societal level (macro level). The assessment schemes require optimization, as has also become clear in other research work (28). It is essential to define and establish objective criteria derived from legislation and applicable across the entire institution.

Finally, the lack of financial resource must be addressed, as this circumstance was mentioned in several interviews.

“[ … ] Currently, protection against violence [ … ] is not a must-have, but rather a nice-to-have [ … ], because it is not financed by the cost bearers.” (ID 5, personal communication, author’s translation)

The cost bearers do not yet provide sufficient funding for prevention activities and there is not enough political interest. The study of political and social issues also receives little attention in literature (25), although literature indicates that political and social approaches must be part of the solution strategies (29). Vrablik et al. propose open communication between justice and healthcare sectors (19). Even though there are no legal requirements for centralized monitoring of violence incidents at the workplace, institutions can use this approach to initiate such communication.

### 4.1 Implications for practice

The results from our interviews provide concrete implications. The study’s findings reveal dynamics that confirm the need to approach effective violence prevention at the micro, meso, and macro levels (19). Although legal requirements for implementing a violence prevention plan have existed at the macro level for several years, the vague wording of these provisions means there is no clear framework within which institutions can operate. Accordingly, these concepts focus primarily on patients and the aggression that may be directed toward them. The perspective of workplace violence is often overlooked.

First, institutions should clarify how they define violence and what objective criteria exist to describe violence. To this end, it is necessary to consider not only the individual micro level but also the meso level to objectively describe incidents of violence. The existing reporting forms should also be reviewed and redesigned for this purpose. This could provide the individuals involved with much greater clarity regarding when an incident is to be considered as an act of violence. Also, this would make it clear when people should seek help. That alone could help to bring cultural change, foster a different attitude toward these incidents, and ultimately lead to better care. Next, the newly introduced definition of violence also serves to provide a more accurate representation of incidents, which is essential for documenting and handling the latter. A structured, concise, and centralized documentation and handling procedures will lead to a reliable data foundation. Fast and user-friendly reporting systems, as well as support from management and supervisors, are considered contributing factors (30). This could serve to identify targeted preventive measures. Clear guidelines and structured documentation can themselves be viewed as preventive measures (31). It also contributes to making violence in the elderly and special needs care sector much more visible. It can thus influence the macro level and help ensure that the issue is prioritized at the political level as well as by funding providers.

### 4.2 Limitations

Like any study, this one is also subject to certain limitations. The selected sample represents only a small part of the subject matter. Participants were purposeful recruited in elderly and special needs care institutions in southern Germany to take part in the study. Additionally, four participants were from institutions in the KompIGA project network. All participants expressed a high level of interest in the topic. This is a sensitive topic. The interviews consist solely of self-reported data. These factors may have resulted in some social desirability bias. Furthermore, only expert perspectives were included. The individuals who work directly with clients were not interviewed. All stakeholders involved in the process should be included, particularly when developing digital applications. It is essential to survey not only those responsible for the process, but above all those who report incidents (i.e., employees or clients). Future studies should include these target groups. Finally, it should be noted that the interviews and subsequent analysis were conducted by a single person. Particular emphasis was placed on ways to support violence prevention programs digitally. The resulting proposed solutions are intended to be usable on a broad user base. There is no focus on the nature and extent of incidents of violence. These could vary significantly among the institutions under consideration.

## 5 Conclusion

This paper provides an exploratory overview of violence prevention activities in elderly and special needs care institutions. It clearly shows that all study participants consider the issue to be urgent and are implementing initial measures to improve protection against violence. However, when conducting the interviews, it became clear that violence is a highly subjective topic and hard to classify, even for experts in this domain. This is necessary for documenting and uncovering the unknown number of violent incidents in this industry. Organizational process development must start at the root, specifically with the definition of violence. Clearly defining what constitutes violence is essential for determining when incidents must be documented and further investigated. This does not mean that subjective perception plays no role, but rather that it must be supported by objective criteria. This way, targeted assistance and prevention services can be implemented and provided. This also provides an opportunity to determine frequencies and assess the resulting workload caused by violence in the workplace. Digital and uniform reporting would also result in incidents being more visible. Given the severely limited resources, these statistics could help to provide arguments to service providers and strengthen prevention efforts. The overarching goal of improving the quality of care and working conditions could thus be achieved. The potential risks for both clients and employees can be better assessed. Including data into risk analysis contributes to a holistic view of violent incidents. In order to develop an application for reporting, documenting, and handling incidents of violence, requirements were identified. These are addressed in the publication “Rethinking Violence Prevention in Elderly and Special Needs Care: Requirements for Digital Solutions” (32).

Finally, need for additional research was uncovered. It should be noted that greater efforts should be made to report violence based on more objective criteria. In addition to defining specific criteria, it is particularly important to examine the impact of violence on employees, clients and other stakeholders. Particularly in view of the already high turnover rates and poor staffing levels, the interrelationships must be examined more closely, and solutions need to be developed. Finally, the frequent mention of structural and, above all, personnel factors lead to interactions between working conditions and acts of violence.

## Data Availability

The raw data supporting the conclusions of this article will be made available by the authors, without undue reservation.

## Footnotes

1 https://gehba.de/; last accessed: 02/19/2026

2 https://kompiga.de/; last accessed: 02/19/2026
