# Supplementary material for: Violence prevention and incident documentation in elderly and special needs care institutions in southern Germany: results from semi-structured interviews
Source: Front Psychiatry. 2026 Oct 21;17:1838036. doi: 10.3389/fpsyt.2026.1838036 (PMC13731392; doi:10.3389/fpsyt.2026.1838036)
Supplement: Supplementary file 1 [file Table1.docx]

Supplementary Material

# 1 Supplementary Tables

**Table S1.** A Set of 33 Species for Phylogenetic Analysis and Tree Constructionn.

| **Number** | **order** | **Family** | **Genus** | **Scientific name** | **GenBank accession number** |
| --- | --- | --- | --- | --- | --- |
| 1 | Rosales | Cannabiaceae | Pteroceltis | *Pteroceltis tatarinowii* | PZ239996 |
| 2 |  |  | Cannabis | *Cannabis sativa* | NC_029855.1 |
| 3 |  |  | Humulus | *Humulus lupulus* | NC_086845.1 |
| 4 |  | Moraceae | Morus | *Morus notabilis* | NC_041177.1 |
| 5 |  |  | Morus | *Morus rubra* | PX233331.1 |
| 6 |  |  | Artocarpus | *Artocarpus heterophyllus* | PQ839730.1 |
| 7 |  |  | Artocarpus | *Artocarpus integer* | PQ839729.1 |
| 8 |  |  | Ficus | *Ficus carica* | NC_077626.1 |
| 9 |  |  | Ficus | *Ficus hirta* | PV472256.1 |
| 10 |  |  | Antiaris | *Antiaris toxicaria* | PX109644.1 |
| 11 |  | Rosaceae | Dasiphora | *Dasiphora fruticosa* | PV591331.1 |
| 12 |  |  | Dasiphora | *Dasiphora glabra* | PV591332.1 |
| 13 |  |  | Fragaria | *Fragaria mandshurica* | NC_065222.1 |
| 14 |  |  | Fragaria | *Fragaria vesca* | NC_065239.1 |
| 15 |  |  | Malus | *Malus delavayi* | PX108894.1 |
| 16 |  |  | Malus | *Malus domestica* | PP493239.1 |
| 17 |  |  | Potentilla | *Potentilla lineata* | PV591330.1 |
| 18 |  |  | Potentilla | *Potentilla micrantha* | NC_062588.1 |
| 19 |  |  | Prunus | *Prunus campanulata* | PQ842642.1 |
| 20 |  |  | Prunus | *Prunus fruticosa* | PQ842643.1 |
| 21 |  |  | Pyrus | *Pyrus calleryana* | PX222707.1 |
| 22 |  |  | Pyrus | *Pyrus pyrifolia* | PX233706.1 |
| 23 |  |  | Rosa | *Rosa chinensis* | NC_065236.1 |
| 24 |  |  | Rosa | *Rosa roxburghii* | PX109643.1 |
| 25 |  |  | Spiraea | *Spiraea chinensis* | OR643883.1-OR643884.1 |
| 26 |  |  | Spiraea | *Spiraea japonica* | OR643885.1-OR643886.1 |
| 27 |  | Rhamnaceae | Ziziphus | *Ziziphus jujuba* | NC_029809.1 |
| 28 |  |  | Ziziphus | *Ziziphus mauritiana* | NC_068745.1 |
| 29 |  |  | Ventilago | *Ventilago leiocarpa* | OQ165322.1-OQ165324.1 |
| 30 |  | Elaeagnaceae | Hippophae | *Hippophae rhamnoides* | PX529389.1 |
| 31 |  |  | Hippophae | *Hippophae tibetana* | PP712939.1 |
| 32 | Fabales | Fabaceae | Glycine | *Glycine max* | NC_020455.1 |
| 33 |  |  | Glycine | *Glycine soja* | NC_039768.1 |

**Table S2.** Analysis of Length and Sequencing Depth at Different Loci in the itochondrial Genome of Pteroceltis tatarinowii .

| **Contig/Node** | **Length (bp)** | **Depth (×)** |
| --- | --- | --- |
| ctg1 | 310,616 | 180 |
| ctg2 | 71,039 | 168 |
| ctg3 | 52,180 | 161 |
| ctg4 | 45,302 | 172 |
| ctg5 | 23,301 | 196 |
| ctg6 | 3,281 | 358 |

**Table S3.** Protein-Coding Genes in the Mitochondrial Genome of *Pteroceltis tatarinowii*.

| **Group of genes** | **Name of genes** |
| --- | --- |
| ATP synthase | *atp*1，*atp*4，*atp*6（×2），*atp*8，*atp*9 |
| NADH dehydrogenase | *nad*1，*nad*2，*nad*3，*nad*4，*nad*4L，*nad*5，*nad*6，*nad*7，*nad*9 |
| Cytochrome *b* | *cob* |
| Cytochrome *c* biogenesis | *ccm*B，*ccm*C，*ccm*FC，*ccm*FN |
| Cytochrome *c* oxidase | *cox*1，*cox*2，*cox*3 |
| Maturases | *mat*R |
| Protein transport subunit | *mtt*B |
| Ribosomal protein large subunit | *rpl*5，*rpl*10，*rpl*16 |
| Ribosomal protein small subunit | *rps*3，*rps*4，*rps*7，*rps*10，*rps*12，*rps*13，*rps*14，*rps*19 |
| Succinate dehydrogenase | *sdh*3，*sdh*4 |
| Ribosomal RNA | *rrn*5，*rrn*18，*rrn*26 |
| Transfer RNA | *trn*C-GCA，*trn*D-GUC，*trn*E-UUC，*trn*F-GAA，*trnf*M-CAU，*trn*G-GCC，*trn*H-GUG，*trn*I-CAU，*trn*K-UUU，*trn*M-CAU，*trn*N-GUU，*trn*P-UGG（×2），*trn*Q-UUG（×2），*trn*S-GCU，*trn*W-CCA，*trn*Y-GUA |
| Note: Numbers in parentheses indicate the copy number of each gene, for example, (×2) represents two copies. | |

**Table S4.** Analysis of Relative Synonymous Codon Usage (RSCU) for Amino Acids in the Mitochondrial Genome of *Pteroceltis tatarinowii*.

| **Amino** | **Codon 1** | **Codon 2** | **Codon 3** | **Codon 4** | **Codon 5** | **Codon 6** |
| --- | --- | --- | --- | --- | --- | --- |
|  | **RSCU** | **RSCU** | **RSCU** | **RSCU** | **RSCU** | **RSCU** |
| Ala | GCU | GCA | GCC | GCG |  |  |
|  | 1.58 | 0.99 | 0.95 | 0.48 |  |  |
| Arg | AGA | CGA | CGU | AGG | CGG | CGC |
|  | 1.44 | 1.32 | 1.23 | 0.7 | 0.69 | 0.61 |
| Asn | AAU | AAC |  |  |  |  |
|  | 1.34 | 0.66 |  |  |  |  |
| Asp | GAU | GAC |  |  |  |  |
|  | 1.39 | 0.61 |  |  |  |  |
| Cys | UGU | UGC |  |  |  |  |
|  | 1.3 | 0.7 |  |  |  |  |
| End | UAA | UGA | UAG |  |  |  |
|  | 1.59 | 0.79 | 0.62 |  |  |  |
| Gln | CAA | CAG |  |  |  |  |
|  | 1.52 | 0.48 |  |  |  |  |
| Glu | GAA | GAG |  |  |  |  |
|  | 1.4 | 0.6 |  |  |  |  |
| Gly | GGA | GGU | GGG | GGC |  |  |
|  | 1.48 | 1.27 | 0.74 | 0.5 |  |  |
| His | CAU | CAC |  |  |  |  |
|  | 1.51 | 0.49 |  |  |  |  |
| Ile | AUU | AUC | AUA |  |  |  |
|  | 1.32 | 0.85 | 0.83 |  |  |  |
| Leu | UUA | UUG | CUU | CUA | CUG | CUC |
|  | 1.41 | 1.23 | 1.23 | 0.97 | 0.59 | 0.57 |
| Lys | AAA | AAG |  |  |  |  |
|  | 1.21 | 0.79 |  |  |  |  |
| Met | AUG |  |  |  |  |  |
|  | 1 |  |  |  |  |  |
| Phe | UUU | UUC |  |  |  |  |
|  | 1.14 | 0.86 |  |  |  |  |
| Pro | CCU | CCA | CCC | CCG |  |  |
|  | 1.55 | 1.09 | 0.76 | 0.6 |  |  |
| Ser | UCU | UCA | AGU | UCC | UCG | AGC |
|  | 1.38 | 1.17 | 1.07 | 1 | 0.79 | 0.6 |
| Thr | ACU | ACC | ACA | ACG |  |  |
|  | 1.41 | 1.01 | 0.99 | 0.59 |  |  |
| Trp | UGG |  |  |  |  |  |
|  | 1 |  |  |  |  |  |
| Tyr | UAU | UAC |  |  |  |  |
|  | 1.53 | 0.47 |  |  |  |  |
| Val | GUU | GUA | GUG | GUC |  |  |
|  | 1.28 | 1.19 | 0.84 | 0.69 |  |  |

**Table S5.** Dispersed repeat sequences in the mitochondrial genome of *Pteroceltis tatarinowii* .

| **ID** | **Length** | **Type** | **The first repeating unit** | | | **The second repeating unit** | | | |
| --- | --- | --- | --- | --- | --- | --- | --- | --- | --- |
|  |  |  | **Position** | **Start** | **End** | **Position** | **Start** | **End** | |
| R1 | 3281 | F | contig1 | 379220 | 382500 | contig1 | 453540 | | 456820 |
| R2 | 756 | P | contig1 | 177657 | 178412 | contig1 | 333053 | | 333808 |
| R3 | 267 | P | contig1 | 271254 | 271520 | contig1 | 332379 | | 332645 |
| R4 | 197 | F | contig1 | 227845 | 228041 | contig1 | 425624 | | 425820 |
| R5 | 169 | F | contig1 | 897 | 1065 | contig1 | 67573 | | 67741 |
| R6 | 148 | F | contig1 | 282715 | 282862 | contig1 | 413743 | | 413890 |
| R7 | 126 | P | contig1 | 291664 | 291789 | contig1 | 502572 | | 502697 |
| R8 | 104 | P | contig1 | 368661 | 368764 | contig1 | 479985 | | 480088 |
| R9 | 94 | P | contig1 | 291800 | 291893 | contig1 | 502476 | | 502569 |
| R10 | 94 | F | contig1 | 286033 | 286126 | contig1 | 333598 | | 333691 |
| R11 | 94 | P | contig1 | 286033 | 286126 | contig1 | 177774 | | 177867 |
| R12 | 94 | P | contig1 | 333598 | 333691 | contig1 | 177774 | | 177867 |
| R13 | 93 | P | contig1 | 353553 | 353645 | contig1 | 398570 | | 398662 |
| R14 | 79 | F | contig1 | 232224 | 232302 | contig1 | 414023 | | 414101 |
| R15 | 78 | F | contig1 | 85113 | 85190 | contig1 | 162506 | | 162583 |
| R16 | 77 | F | contig1 | 332277 | 332353 | contig1 | 451597 | | 451673 |
| R17 | 77 | F | contig1 | 282637 | 282713 | contig1 | 413665 | | 413741 |
| R18 | 75 | P | contig1 | 353727 | 353801 | contig1 | 398421 | | 398495 |
| R19 | 75 | F | contig1 | 6693 | 6767 | contig1 | 368941 | | 369015 |
| R20 | 74 | F | contig1 | 178514 | 178587 | contig1 | 271497 | | 271570 |
| R21 | 73 | P | contig1 | 291690 | 291762 | contig1 | 496476 | | 496548 |
| R22 | 73 | P | contig1 | 291690 | 291762 | contig1 | 502599 | | 502671 |
| R23 | 73 | F | contig1 | 496476 | 496548 | contig1 | 502599 | | 502671 |
| R24 | 71 | P | contig1 | 368606 | 368676 | contig1 | 88613 | 88683 | |
| R25 | 70 | P | contig1 | 307326 | 307395 | contig1 | 88595 | 88664 | |
| R26 | 67 | F | contig1 | 137856 | 137922 | contig1 | 489833 | 489899 | |
| R27 | 67 | F | contig1 | 98386 | 98452 | contig1 | 414103 | 414169 | |
| R28 | 66 | F | contig1 | 6319 | 6384 | contig1 | 452719 | 452784 | |
| R29 | 65 | P | contig1 | 316880 | 316944 | contig1 | 447834 | 447898 | |
| R30 | 65 | P | contig1 | 246855 | 246919 | contig1 | 436180 | 436244 | |
| R31 | 65 | F | contig1 | 97184 | 97248 | contig1 | 384491 | 384555 | |
| R32 | 63 | F | contig1 | 232254 | 232316 | contig1 | 233285 | 233347 | |
| R33 | 61 | P | contig1 | 291383 | 291443 | contig1 | 502918 | 502978 | |
| R34 | 57 | P | contig1 | 246752 | 246808 | contig1 | 436299 | 436355 | |
| R35 | 57 | P | contig1 | 123376 | 123432 | contig1 | 269574 | 269630 | |
| R36 | 55 | F | contig1 | 25645 | 25699 | contig1 | 178503 | 178557 | |
| R37 | 54 | F | contig1 | 96078 | 96131 | contig1 | 390232 | 390285 | |
| R38 | 54 | F | contig1 | 36791 | 36844 | contig1 | 97805 | 97858 | |
| R39 | 52 | P | contig1 | 88613 | 88664 | contig1 | 307326 | 307377 | |
| R40 | 52 | P | contig1 | 88613 | 88664 | contig1 | 368625 | 368676 | |
| R41 | 52 | F | contig1 | 307326 | 307377 | contig1 | 368625 | 368676 | |
| R42 | 52 | P | contig1 | 94223 | 94274 | contig1 | 183272 | 183323 | |
| R43 | 51 | F | contig1 | 398326 | 398376 | contig1 | 450117 | 450167 | |
| R44 | 51 | P | contig1 | 88548 | 88598 | contig1 | 183095 | 183145 | |
| R45 | 50 | F | contig1 | 230712 | 230761 | contig1 | 269583 | 269632 | |
| R46 | 49 | F | contig1 | 232254 | 232302 | contig1 | 233285 | 233333 | |
| R47 | 49 | F | contig1 | 232254 | 232302 | contig1 | 414053 | 414101 | |
| R48 | 49 | F | contig1 | 233285 | 233333 | contig1 | 414053 | 414101 | |
| R49 | 48 | P | contig1 | 123376 | 123423 | contig1 | 230712 | 230759 | |
| R50 | 48 | P | contig1 | 123376 | 123423 | contig1 | 269583 | 269630 | |
| R51 | 48 | F | contig1 | 230712 | 230759 | contig1 | 269583 | 269630 | |
| R52 | 48 | P | contig1 | 94033 | 94080 | contig1 | 292633 | 292680 | |
| R53 | 46 | P | contig1 | 314592 | 314637 | contig1 | 88145 | 88190 | |
| R54 | 46 | P | contig1 | 66228 | 66273 | contig1 | 66228 | 66273 | |
| R55 | 45 | P | contig1 | 135160 | 135204 | contig1 | 299039 | 299083 | |
| R56 | 45 | F | contig1 | 55507 | 55551 | contig1 | 289699 | 289743 | |
| R57 | 44 | P | contig1 | 246945 | 246988 | contig1 | 436111 | 436154 | |
| R58 | 44 | F | contig1 | 193127 | 193170 | contig1 | 420159 | 420202 | |
| R59 | 44 | F | contig1 | 25656 | 25699 | contig1 | 178514 | 178557 | |
| R60 | 44 | F | contig1 | 25656 | 25699 | contig1 | 271497 | 271540 | |
| R61 | 44 | F | contig1 | 178514 | 178557 | contig1 | 271497 | 271540 | |
| R62 | 44 | F | contig1 | 1067 | 1110 | contig1 | 67743 | 67786 | |
| R63 | 43 | F | contig1 | 255797 | 255839 | contig1 | 325645 | 325687 | |
| R64 | 43 | P | contig1 | 246566 | 246608 | contig1 | 436497 | 436539 | |
| R65 | 43 | F | contig1 | 164672 | 164714 | contig1 | 307393 | 307435 | |
| R66 | 42 | P | contig1 | 246675 | 246716 | contig1 | 436391 | 436432 | |
| R67 | 42 | F | contig1 | 95860 | 95901 | contig1 | 452336 | 452377 | |
| R68 | 42 | F | contig1 | 85558 | 85599 | contig1 | 366304 | 366345 | |
| R69 | 42 | P | contig1 | 95284 | 95325 | contig1 | 84381 | 84422 | |
| R70 | 41 | F | contig1 | 234905 | 234945 | contig1 | 503585 | 503625 | |
| R71 | 41 | P | contig1 | 135977 | 136017 | contig1 | 474563 | 474603 | |
| R72 | 41 | F | contig1 | 36833 | 36873 | contig1 | 189325 | 189365 | |
| R73 | 40 | F | contig1 | 342422 | 342461 | contig1 | 342461 | 342500 | |
| R74 | 39 | F | contig1 | 398278 | 398316 | contig1 | 450069 | 450107 | |
| R75 | 39 | P | contig1 | 189233 | 189271 | contig1 | 269056 | 269094 | |
| R76 | 39 | F | contig1 | 62961 | 62999 | contig1 | 307945 | 307983 | |
| R77 | 37 | F | contig1 | 188974 | 189010 | contig1 | 428940 | 428976 | |
| R78 | 36 | F | contig1 | 282931 | 282966 | contig1 | 415112 | 415147 | |
| R79 | 36 | P | contig1 | 325613 | 325648 | contig1 | 255834 | 255869 | |
| R80 | 35 | F | contig1 | 81435 | 81469 | contig1 | 84383 | 84417 | |
| R81 | 35 | P | contig1 | 81435 | 81469 | contig1 | 95289 | 95323 | |
| R82 | 35 | P | contig1 | 84383 | 84417 | contig1 | 95289 | 95323 | |
| R83 | 34 | P | contig1 | 353759 | 353792 | contig1 | 398430 | 398463 | |
| R84 | 34 | P | contig1 | 353759 | 353792 | contig1 | 450237 | 450270 | |
| R85 | 34 | F | contig1 | 398430 | 398463 | contig1 | 450237 | 450270 | |
| R86 | 34 | F | contig1 | 341069 | 341102 | contig1 | 467444 | 467477 | |
| R87 | 34 | P | contig1 | 480415 | 480448 | contig1 | 164457 | 164490 | |
| R88 | 34 | P | contig1 | 127248 | 127281 | contig1 | 247620 | 247653 | |
| R89 | 34 | F | contig1 | 126350 | 126383 | contig1 | 307592 | 307625 | |
| R90 | 34 | F | contig1 | 125291 | 125324 | contig1 | 480151 | 480184 | |
| R91 | 34 | F | contig1 | 83073 | 83106 | contig1 | 184022 | 184055 | |
| R92 | 34 | P | contig1 | 2762 | 2795 | contig1 | 491303 | 491336 | |
| R93 | 33 | F | contig1 | 414011 | 414043 | contig1 | 415158 | 415190 | |
| R94 | 33 | P | contig1 | 347396 | 347428 | contig1 | 451380 | 451412 | |
| R95 | 33 | F | contig1 | 284982 | 285014 | contig1 | 285735 | 285767 | |
| R96 | 33 | F | contig1 | 283460 | 283492 | contig1 | 495718 | 495750 | |
| R97 | 33 | F | contig1 | 232177 | 232209 | contig1 | 413976 | 414008 | |
| R98 | 33 | F | contig1 | 24760 | 24792 | contig1 | 213808 | 213840 | |
| R99 | 32 | F | contig1 | 307547 | 307578 | contig1 | 368738 | 368769 | |
| R100 | 32 | P | contig1 | 291631 | 291662 | contig1 | 502699 | 502730 | |
| R101 | 32 | P | contig1 | 246719 | 246750 | contig1 | 436357 | 436388 | |
| R102 | 32 | F | contig1 | 85080 | 85111 | contig1 | 162473 | 162504 | |
| R103 | 32 | F | contig1 | 36764 | 36795 | contig1 | 230679 | 230710 | |
| R104 | 32 | F | contig1 | 14475 | 14506 | contig1 | 17242 | 17273 | |
| R105 | 31 | F | contig1 | 411548 | 411578 | contig1 | 412830 | 412860 | |
| R106 | 31 | P | contig1 | 296707 | 296737 | contig1 | 297161 | 297191 | |
| R107 | 31 | P | contig1 | 296388 | 296418 | contig1 | 397761 | 397791 | |
| R108 | 31 | P | contig1 | 316584 | 316614 | contig1 | 285692 | 285722 | |
| R109 | 31 | P | contig1 | 193360 | 193390 | contig1 | 302554 | 302584 | |
| R110 | 31 | F | contig1 | 170502 | 170532 | contig1 | 170545 | 170575 | |
| R111 | 31 | F | contig1 | 96033 | 96063 | contig1 | 390187 | 390217 | |
| R112 | 30 | P | contig1 | 467288 | 467317 | contig1 | 467288 | 467317 | |
| R113 | 30 | P | contig1 | 502790 | 502819 | contig1 | 291542 | 291571 | |
| R114 | 30 | P | contig1 | 346923 | 346952 | contig1 | 176844 | 176873 | |
| R115 | 30 | F | contig1 | 121339 | 121368 | contig1 | 121446 | 121475 | |
| R116 | 30 | P | contig1 | 104439 | 104468 | contig1 | 106163 | 106192 | |
| R117 | 30 | P | contig1 | 408462 | 408491 | contig1 | 99185 | 99214 | |

**Table S6.** Homologous DNA fragments in the *Pteroceltis tatarinowii* mitochondrial genome.

| **Number** | **Mitochondrial genome** | **% Identity** | **Alignment Length** | **Mismatches** | **Gap Openings** | **Alignment start (chloroplast genome)** | **Alignment end (chloroplast genome)** | **Alignment start (mitochondrial genome)** | **Alignment end (mitochondrial genome)** | **E-value** | **Bit Score** | **MTPT annotation** |
| --- | --- | --- | --- | --- | --- | --- | --- | --- | --- | --- | --- | --- |
| MTPT1 | *Pteroceltis tatarinowii* | 100 | 29 | 0 | 0 | 11328 | 11356 | 340746 | 340718 | 2.81E-06 | 54.7 | partial atpA |
| MTPT2 | *Pteroceltis tatarinowii* | 98.78 | 82 | 1 | 0 | 37 | 118 | 480821 | 480740 | 4.50E-34 | 147 | complete trnH-GUG |
| MTPT3 | *Pteroceltis tatarinowii* | 97.973 | 148 | 3 | 0 | 40452 | 40599 | 491474 | 491327 | 1.99E-67 | 257 | partial psaB |
| MTPT4 | *Pteroceltis tatarinowii* | 96.296 | 81 | 2 | 1 | 112224 | 112303 | 423156 | 423076 | 1.26E-29 | 132 | complete trnN-GUU |
|  | *Pteroceltis tatarinowii* | 96.296 | 81 | 2 | 1 | 133861 | 133940 | 423076 | 423156 | 1.26E-29 | 132 | complete trnN-GUU |
| MTPT5 | *Pteroceltis tatarinowii* | 94.624 | 93 | 2 | 3 | 32222 | 32311 | 72033 | 71941 | 2.10E-32 | 141 | complete trnD-GUC |
| MTPT6 | *Pteroceltis tatarinowii* | 92.405 | 79 | 6 | 0 | 54735 | 54813 | 183839 | 183917 | 4.57E-24 | 113 | complete trnM-CAU |
| MTPT7 | *Pteroceltis tatarinowii* | 91.892 | 148 | 11 | 1 | 36970 | 37116 | 341474 | 341621 | 7.33E-52 | 206 | partial psbC |

**Table S7.** SSRs in the mitochondrial genome of *Pteroceltis tatarinowii* .

| **Mitochondrial genome** | **SSR nr.ome** | **SSR type** | **SSR** | **size** | **start** | **end** |
| --- | --- | --- | --- | --- | --- | --- |
| *Pteroceltis tatarinowii* | 1 | p4 | (TTTC)3 | 12 | 2621 | 2632 |
| *Pteroceltis tatarinowii* | 2 | p3 | (AGA)4 | 12 | 2732 | 2743 |
| *Pteroceltis tatarinowii* | 3 | p2 | (GA)6 | 12 | 6520 | 6531 |
| *Pteroceltis tatarinowii* | 4 | p1 | (T)10 | 10 | 10106 | 10115 |
| *Pteroceltis tatarinowii* | 5 | p1 | (T)12 | 12 | 12910 | 12921 |
| *Pteroceltis tatarinowii* | 6 | p1 | (G)11 | 11 | 17484 | 17494 |
| *Pteroceltis tatarinowii* | 7 | p1 | (T)10 | 10 | 20454 | 20463 |
| *Pteroceltis tatarinowii* | 8 | p4 | (TATC)3 | 12 | 21065 | 21076 |
| *Pteroceltis tatarinowii* | 9 | p4 | (ACGA)3 | 12 | 23759 | 23770 |
| *Pteroceltis tatarinowii* | 10 | p5 | (CTTTA)3 | 15 | 24727 | 24741 |
| *Pteroceltis tatarinowii* | 11 | p4 | (AATC)3 | 12 | 25990 | 26001 |
| *Pteroceltis tatarinowii* | 12 | p3 | (AGG)5 | 15 | 26878 | 26892 |
| *Pteroceltis tatarinowii* | 13 | p2 | (GA)5 | 10 | 27014 | 27023 |
| *Pteroceltis tatarinowii* | 14 | p3 | (TTC)4 | 12 | 33166 | 33177 |
| *Pteroceltis tatarinowii* | 15 | p4 | (ATTC)3 | 12 | 33316 | 33327 |
| *Pteroceltis tatarinowii* | 16 | p1 | (A)12 | 12 | 36527 | 36538 |
| *Pteroceltis tatarinowii* | 17 | p1 | (T)10 | 10 | 38174 | 38183 |
| *Pteroceltis tatarinowii* | 18 | p4 | (TTCT)4 | 16 | 52321 | 52336 |
| *Pteroceltis tatarinowii* | 19 | p4 | (TTCT)3 | 12 | 53374 | 53385 |
| *Pteroceltis tatarinowii* | 20 | p4 | (CGGC)3 | 12 | 60676 | 60687 |
| *Pteroceltis tatarinowii* | 21 | p1 | (A)10 | 10 | 63845 | 63854 |
| *Pteroceltis tatarinowii* | 22 | p1 | (A)10 | 10 | 71114 | 71123 |
| *Pteroceltis tatarinowii* | 23 | p1 | (A)12 | 12 | 71551 | 71562 |
| *Pteroceltis tatarinowii* | 24 | p1 | (T)10 | 10 | 71578 | 71587 |
| *Pteroceltis tatarinowii* | 25 | p1 | (T)10 | 10 | 72331 | 72340 |
| *Pteroceltis tatarinowii* | 26 | p4 | (ATCT)3 | 12 | 72451 | 72462 |
| *Pteroceltis tatarinowii* | 27 | p1 | (A)10 | 10 | 72586 | 72595 |
| *Pteroceltis tatarinowii* | 28 | p2 | (CT)5 | 10 | 73784 | 73793 |
| *Pteroceltis tatarinowii* | 29 | p3 | (GAT)4 | 12 | 74073 | 74084 |
| *Pteroceltis tatarinowii* | 30 | p4 | (GATG)3 | 12 | 76206 | 76217 |
| *Pteroceltis tatarinowii* | 31 | p4 | (ATAA)3 | 12 | 77436 | 77447 |
| *Pteroceltis tatarinowii* | 32 | p1 | (T)10 | 10 | 83200 | 83209 |
| *Pteroceltis tatarinowii* | 33 | p4 | (ATGG)3 | 12 | 84168 | 84179 |
| *Pteroceltis tatarinowii* | 34 | p5 | (TCTTC)3 | 15 | 84198 | 84212 |
| *Pteroceltis tatarinowii* | 35 | p4 | (CTTT)3 | 12 | 84233 | 84244 |
| *Pteroceltis tatarinowii* | 36 | p1 | (T)11 | 11 | 84972 | 84982 |
| *Pteroceltis tatarinowii* | 37 | p2 | (AG)5 | 10 | 85239 | 85248 |
| *Pteroceltis tatarinowii* | 38 | p2 | (GA)6 | 12 | 87068 | 87079 |
| *Pteroceltis tatarinowii* | 39 | p4 | (CCGC)3 | 12 | 88979 | 88990 |
| *Pteroceltis tatarinowii* | 40 | p4 | (CTAG)3 | 12 | 89604 | 89615 |
| *Pteroceltis tatarinowii* | 41 | p4 | (GAAG)3 | 12 | 92968 | 92979 |
| *Pteroceltis tatarinowii* | 42 | p5 | (GTAAT)3 | 15 | 94606 | 94620 |
| *Pteroceltis tatarinowii* | 43 | p1 | (G)11 | 11 | 95723 | 95733 |
| *Pteroceltis tatarinowii* | 44 | p1 | (A)10 | 10 | 96522 | 96531 |
| *Pteroceltis tatarinowii* | 45 | p4 | (AGGC)3 | 12 | 99406 | 99417 |
| *Pteroceltis tatarinowii* | 46 | p1 | (T)10 | 10 | 104567 | 104576 |
| *Pteroceltis tatarinowii* | 47 | p2 | (CT)5 | 10 | 106021 | 106030 |
| *Pteroceltis tatarinowii* | 48 | p4 | (TCAT)3 | 12 | 108041 | 108052 |
| *Pteroceltis tatarinowii* | 49 | p2 | (CT)6 | 12 | 110006 | 110017 |
| *Pteroceltis tatarinowii* | 50 | p2 | (CA)5 | 10 | 120351 | 120360 |
| *Pteroceltis tatarinowii* | 51 | p4 | (ATAA)3 | 12 | 122080 | 122091 |
| *Pteroceltis tatarinowii* | 52 | p2 | (TA)6 | 12 | 129789 | 129800 |
| *Pteroceltis tatarinowii* | 53 | p2 | (CT)5 | 10 | 130072 | 130081 |
| *Pteroceltis tatarinowii* | 54 | p6 | (TCTTTC)3 | 18 | 133751 | 133768 |
| *Pteroceltis tatarinowii* | 55 | p4 | (GGCT)3 | 12 | 143597 | 143608 |
| *Pteroceltis tatarinowii* | 56 | p4 | (TGAA)3 | 12 | 146885 | 146896 |
| *Pteroceltis tatarinowii* | 57 | p4 | (AAAG)3 | 12 | 156272 | 156283 |
| *Pteroceltis tatarinowii* | 58 | p4 | (GCTT)3 | 12 | 164891 | 164902 |
| *Pteroceltis tatarinowii* | 59 | p4 | (AGAA)3 | 12 | 165569 | 165580 |
| *Pteroceltis tatarinowii* | 60 | p4 | (GGAA)3 | 12 | 167743 | 167754 |
| *Pteroceltis tatarinowii* | 61 | p2 | (TA)5 | 10 | 169699 | 169708 |
| *Pteroceltis tatarinowii* | 62 | p4 | (ATAA)3 | 12 | 177683 | 177694 |
| *Pteroceltis tatarinowii* | 63 | p2 | (TA)5 | 10 | 178865 | 178874 |
| *Pteroceltis tatarinowii* | 64 | p2 | (AT)5 | 10 | 178875 | 178884 |
| *Pteroceltis tatarinowii* | 65 | p4 | (TTCT)3 | 12 | 180160 | 180171 |
| *Pteroceltis tatarinowii* | 66 | p4 | (AATA)3 | 12 | 180847 | 180858 |
| *Pteroceltis tatarinowii* | 67 | p3 | (TTA)4 | 12 | 183012 | 183023 |
| *Pteroceltis tatarinowii* | 68 | p4 | (ATTC)3 | 12 | 183341 | 183352 |
| *Pteroceltis tatarinowii* | 69 | p2 | (GA)5 | 10 | 184135 | 184144 |
| *Pteroceltis tatarinowii* | 70 | p1 | (T)13 | 13 | 184543 | 184555 |
| *Pteroceltis tatarinowii* | 71 | p2 | (TA)5 | 10 | 185397 | 185406 |
| *Pteroceltis tatarinowii* | 72 | p4 | (TTTC)3 | 12 | 185496 | 185507 |
| *Pteroceltis tatarinowii* | 73 | p1 | (T)12 | 12 | 185700 | 185711 |
| *Pteroceltis tatarinowii* | 74 | p2 | (AT)5 | 10 | 186524 | 186533 |
| *Pteroceltis tatarinowii* | 75 | p1 | (T)10 | 10 | 186868 | 186877 |
| *Pteroceltis tatarinowii* | 76 | p4 | (ATAA)3 | 12 | 189426 | 189437 |
| *Pteroceltis tatarinowii* | 77 | p4 | (TAGC)3 | 12 | 189456 | 189467 |
| *Pteroceltis tatarinowii* | 78 | p4 | (ACCG)3 | 12 | 191756 | 191767 |
| *Pteroceltis tatarinowii* | 79 | p4 | (TCAT)3 | 12 | 192814 | 192825 |
| *Pteroceltis tatarinowii* | 80 | p2 | (AT)8 | 16 | 203028 | 203043 |
| *Pteroceltis tatarinowii* | 81 | p6 | (ATATAA)3 | 18 | 203040 | 203057 |
| *Pteroceltis tatarinowii* | 82 | p2 | (TC)6 | 12 | 209508 | 209519 |
| *Pteroceltis tatarinowii* | 83 | p2 | (AG)5 | 10 | 209535 | 209544 |
| *Pteroceltis tatarinowii* | 84 | p3 | (TGT)4 | 12 | 211208 | 211219 |
| *Pteroceltis tatarinowii* | 85 | p1 | (C)10 | 10 | 215389 | 215398 |
| *Pteroceltis tatarinowii* | 86 | p5 | (CAGAC)3 | 15 | 217016 | 217030 |
| *Pteroceltis tatarinowii* | 87 | p3 | (GCT)4 | 12 | 219109 | 219120 |
| *Pteroceltis tatarinowii* | 88 | p2 | (TC)5 | 10 | 219719 | 219728 |
| *Pteroceltis tatarinowii* | 89 | p3 | (AGG)4 | 12 | 221065 | 221076 |
| *Pteroceltis tatarinowii* | 90 | p4 | (TTTC)3 | 12 | 222677 | 222688 |
| *Pteroceltis tatarinowii* | 91 | p4 | (AATT)3 | 12 | 223703 | 223714 |
| *Pteroceltis tatarinowii* | 92 | p4 | (GACC)3 | 12 | 226028 | 226039 |
| *Pteroceltis tatarinowii* | 93 | p2 | (TA)6 | 12 | 231333 | 231344 |
| *Pteroceltis tatarinowii* | 94 | p2 | (TC)5 | 10 | 235162 | 235171 |
| *Pteroceltis tatarinowii* | 95 | p2 | (TG)5 | 10 | 239793 | 239802 |
| *Pteroceltis tatarinowii* | 96 | p4 | (ATCT)3 | 12 | 242864 | 242875 |
| *Pteroceltis tatarinowii* | 97 | p2 | (TC)6 | 12 | 244415 | 244426 |
| *Pteroceltis tatarinowii* | 98 | p4 | (TTTG)3 | 12 | 246388 | 246399 |
| *Pteroceltis tatarinowii* | 99 | p2 | (GA)5 | 10 | 246773 | 246782 |
| *Pteroceltis tatarinowii* | 100 | p2 | (AT)10 | 20 | 247385 | 247404 |
| *Pteroceltis tatarinowii* | 101 | p1 | (T)10 | 10 | 259208 | 259217 |
| *Pteroceltis tatarinowii* | 102 | p1 | (A)11 | 11 | 267745 | 267755 |
| *Pteroceltis tatarinowii* | 103 | p4 | (GAGC)3 | 12 | 269116 | 269127 |
| *Pteroceltis tatarinowii* | 104 | p1 | (T)10 | 10 | 270109 | 270118 |
| *Pteroceltis tatarinowii* | 105 | p1 | (T)10 | 10 | 272436 | 272445 |
| *Pteroceltis tatarinowii* | 106 | p5 | (AATAG)3 | 15 | 273452 | 273466 |
| *Pteroceltis tatarinowii* | 107 | p4 | (TGAA)3 | 12 | 275323 | 275334 |
| *Pteroceltis tatarinowii* | 108 | p1 | (T)10 | 10 | 276701 | 276710 |
| *Pteroceltis tatarinowii* | 109 | p4 | (AAGA)3 | 12 | 280076 | 280087 |
| *Pteroceltis tatarinowii* | 110 | p1 | (T)11 | 11 | 280927 | 280937 |
| *Pteroceltis tatarinowii* | 111 | p2 | (AT)6 | 12 | 282479 | 282490 |
| *Pteroceltis tatarinowii* | 112 | p4 | (AAAG)3 | 12 | 289349 | 289360 |
| *Pteroceltis tatarinowii* | 113 | p1 | (A)11 | 11 | 292355 | 292365 |
| *Pteroceltis tatarinowii* | 114 | p2 | (AT)5 | 10 | 295251 | 295260 |
| *Pteroceltis tatarinowii* | 115 | p3 | (ATT)4 | 12 | 295263 | 295274 |
| *Pteroceltis tatarinowii* | 116 | p2 | (AT)5 | 10 | 295278 | 295287 |
| *Pteroceltis tatarinowii* | 117 | p3 | (AAG)4 | 12 | 298251 | 298262 |
| *Pteroceltis tatarinowii* | 118 | p1 | (A)11 | 11 | 303627 | 303637 |
| *Pteroceltis tatarinowii* | 119 | p1 | (C)10 | 10 | 304339 | 304348 |
| *Pteroceltis tatarinowii* | 120 | p4 | (ATAC)3 | 12 | 304351 | 304362 |
| *Pteroceltis tatarinowii* | 121 | p4 | (TTTC)3 | 12 | 304364 | 304375 |
| *Pteroceltis tatarinowii* | 122 | p4 | (AATG)3 | 12 | 306530 | 306541 |
| *Pteroceltis tatarinowii* | 123 | p1 | (T)11 | 11 | 307842 | 307852 |
| *Pteroceltis tatarinowii* | 124 | p1 | (T)10 | 10 | 316306 | 316315 |
| *Pteroceltis tatarinowii* | 125 | p1 | (A)10 | 10 | 317728 | 317737 |
| *Pteroceltis tatarinowii* | 126 | p1 | (T)11 | 11 | 322089 | 322099 |
| *Pteroceltis tatarinowii* | 127 | p3 | (TCA)5 | 15 | 326693 | 326707 |
| *Pteroceltis tatarinowii* | 128 | p4 | (TTAT)3 | 12 | 333771 | 333782 |
| *Pteroceltis tatarinowii* | 129 | p2 | (CT)5 | 10 | 337456 | 337465 |
| *Pteroceltis tatarinowii* | 130 | p2 | (AT)10 | 20 | 341650 | 341669 |
| *Pteroceltis tatarinowii* | 131 | p4 | (AAGG)3 | 12 | 341727 | 341738 |
| *Pteroceltis tatarinowii* | 132 | p3 | (TAA)4 | 12 | 343189 | 343200 |
| *Pteroceltis tatarinowii* | 133 | p1 | (T)11 | 11 | 349900 | 349910 |
| *Pteroceltis tatarinowii* | 134 | p3 | (TCC)4 | 12 | 352106 | 352117 |
| *Pteroceltis tatarinowii* | 135 | p4 | (AAGA)4 | 16 | 360769 | 360784 |
| *Pteroceltis tatarinowii* | 136 | p1 | (T)14 | 14 | 363725 | 363738 |
| *Pteroceltis tatarinowii* | 137 | p5 | (CTAGT)3 | 15 | 368283 | 368297 |
| *Pteroceltis tatarinowii* | 138 | p4 | (AATG)3 | 12 | 368889 | 368900 |
| *Pteroceltis tatarinowii* | 139 | p2 | (AG)5 | 10 | 370057 | 370066 |
| *Pteroceltis tatarinowii* | 140 | p4 | (GAAA)3 | 12 | 379063 | 379074 |
| *Pteroceltis tatarinowii* | 141 | p2 | (TA)8 | 16 | 380107 | 380122 |
| *Pteroceltis tatarinowii* | 142 | p3 | (TTA)4 | 12 | 386256 | 386267 |
| *Pteroceltis tatarinowii* | 143 | p4 | (GTGA)3 | 12 | 387668 | 387679 |
| *Pteroceltis tatarinowii* | 144 | p2 | (AT)5 | 10 | 391843 | 391852 |
| *Pteroceltis tatarinowii* | 145 | p4 | (CTTT)3 | 12 | 392034 | 392045 |
| *Pteroceltis tatarinowii* | 146 | p1 | (A)14 | 14 | 396199 | 396212 |
| *Pteroceltis tatarinowii* | 147 | p1 | (A)11 | 11 | 396289 | 396299 |
| *Pteroceltis tatarinowii* | 148 | p4 | (TAAG)3 | 12 | 397210 | 397221 |
| *Pteroceltis tatarinowii* | 149 | p1 | (T)12 | 12 | 399452 | 399463 |
| *Pteroceltis tatarinowii* | 150 | p2 | (AT)6 | 12 | 399469 | 399480 |
| *Pteroceltis tatarinowii* | 151 | p4 | (GTTA)3 | 12 | 400744 | 400755 |
| *Pteroceltis tatarinowii* | 152 | p1 | (T)11 | 11 | 405480 | 405490 |
| *Pteroceltis tatarinowii* | 153 | p1 | (T)10 | 10 | 406066 | 406075 |
| *Pteroceltis tatarinowii* | 154 | p1 | (T)12 | 12 | 415866 | 415877 |
| *Pteroceltis tatarinowii* | 155 | p1 | (T)11 | 11 | 419276 | 419286 |
| *Pteroceltis tatarinowii* | 156 | p1 | (T)10 | 10 | 420912 | 420921 |
| *Pteroceltis tatarinowii* | 157 | p4 | (TGAC)3 | 12 | 421968 | 421979 |
| *Pteroceltis tatarinowii* | 158 | p4 | (TTCA)3 | 12 | 423662 | 423673 |
| *Pteroceltis tatarinowii* | 159 | p1 | (A)11 | 11 | 430802 | 430812 |
| *Pteroceltis tatarinowii* | 160 | p2 | (TC)5 | 10 | 436325 | 436334 |
| *Pteroceltis tatarinowii* | 161 | p2 | (TA)5 | 10 | 439801 | 439810 |
| *Pteroceltis tatarinowii* | 162 | p2 | (AT)5 | 10 | 445705 | 445714 |
| *Pteroceltis tatarinowii* | 163 | p1 | (T)10 | 10 | 448666 | 448675 |
| *Pteroceltis tatarinowii* | 164 | p5 | (ATGCT)3 | 15 | 451338 | 451352 |
| *Pteroceltis tatarinowii* | 165 | p1 | (C)11 | 11 | 451742 | 451752 |
| *Pteroceltis tatarinowii* | 166 | p2 | (TA)6 | 12 | 452235 | 452246 |
| *Pteroceltis tatarinowii* | 167 | p3 | (TAA)4 | 12 | 453510 | 453521 |
| *Pteroceltis tatarinowii* | 168 | p1 | (T)10 | 10 | 453532 | 453541 |
| *Pteroceltis tatarinowii* | 169 | p2 | (TA)8 | 16 | 454427 | 454442 |
| *Pteroceltis tatarinowii* | 170 | p3 | (GAA)4 | 12 | 463423 | 463434 |
| *Pteroceltis tatarinowii* | 171 | p4 | (TTTG)3 | 12 | 467094 | 467105 |
| *Pteroceltis tatarinowii* | 172 | p1 | (T)10 | 10 | 467529 | 467538 |
| *Pteroceltis tatarinowii* | 173 | p1 | (A)10 | 10 | 472844 | 472853 |
| *Pteroceltis tatarinowii* | 174 | p4 | (TCTA)3 | 12 | 475484 | 475495 |
| *Pteroceltis tatarinowii* | 175 | p4 | (AAAG)3 | 12 | 478591 | 478602 |
| *Pteroceltis tatarinowii* | 176 | p4 | (TTAA)3 | 12 | 478670 | 478681 |
| *Pteroceltis tatarinowii* | 177 | p4 | (AAGA)3 | 12 | 479673 | 479684 |
| *Pteroceltis tatarinowii* | 178 | p4 | (GATA)4 | 16 | 479831 | 479846 |
| *Pteroceltis tatarinowii* | 179 | p2 | (CT)5 | 10 | 483506 | 483515 |
| *Pteroceltis tatarinowii* | 180 | p4 | (TTCC)3 | 12 | 499543 | 499554 |
| *Pteroceltis tatarinowii* | 181 | p4 | (ATCA)4 | 16 | 504081 | 504096 |
| *Pteroceltis tatarinowii* | 182 | p4 | (GCCT)3 | 12 | 506829 | 506840 |
